# From *Spirulina platensis* to Nanomaterials: A Comparative Study of AgNPs Obtained from Two Extracts

**DOI:** 10.3390/nano15181392

**Published:** 2025-09-10

**Authors:** Alexandra Ivanova, Mina Todorova, Dimitar Petrov, Zhana Petkova, Olga Teneva, Ginka Antova, Maria Angelova-Romova, Velichka Yanakieva, Slava Tsoneva, Vera Gledacheva, Krastena Nikolova, Daniela Karashanova, Stoyanka Nikolova

**Affiliations:** 1 Department of Organic Chemistry, Faculty of Chemistry, University of Plovdiv, 4000 Plovdiv, Bulgaria; ivanova.aleksandra@uni-plovdiv.bg (A.I.); minatodorova@uni-plovdiv.bg (M.T.); 2 Department of Physical Chemistry, Faculty of Chemistry, University of Plovdiv, 4000 Plovdiv, Bulgaria; petrov_d@uni-plovdiv.bg; 3 Department of Chemical Technology, Faculty of Chemistry, University of Plovdiv, 4000 Plovdiv, Bulgaria; zhanapetkova@uni-plovdiv.bg (Z.P.); olga@uni-plovdiv.bg (O.T.); ginant@uni-plovdiv.bg (G.A.); maioan@uni-plovdiv.bg (M.A.-R.); 4 Department of Microbiology, Technological Faculty, University of Food Technologies, 4002 Plovdiv, Bulgaria; yanakieva_vili@abv.bg; 5 Department of Analytical Chemistry and Computer Chemistry, University of Plovdiv, 4000 Plovdiv, Bulgaria; slava.tsoneva@uni-plovdiv.bg; 6 Department of Medical Physics and Biophysics, Faculty of Pharmacy, Medical University of Plovdiv, 4002 Plovdiv, Bulgaria; vera.gledacheva@mu-plovdiv.bg; 7 Department of Physics and Biophysics, Faculty of Pharmacy, Medical University of Varna, 84 Tzar Osvoboditel, 9000 Varna, Bulgaria; krastena.nikolova@mu-varna.bg; 8 ”Acad. E. Djakov” Institute of Electronics, Bulgarian Academy of Sciences, 1784 Sofia, Bulgaria; adi@iomt.bas.bg

**Keywords:** *Spirulina platensis*, silver nanoparticles, tocopherols, antimicrobial, anti-inflammatory activity

## Abstract

This study presents the synthesis and characterization of silver nanoparticles (AgNPs) using two *Spirulina platensis* extracts: one of them cultivated in a bioreactor in Bulgaria (near Varvara village), and the other one from the local market in Bulgaria (Dragon Superfoods). To assess their properties and stability, ATR-FTIR, TEM (Transmission Electron Microscopy) images, and zeta potential were used. Chemical content of the extracts and AgNPs obtained were assessed, as well as their antimicrobial and anti-inflammatory activities. We found that the extracts’ origin significantly influenced nanoparticle morphology, surface charge, and bioactivity. AgNPs were spherical and different in size from Bioreactor 4–8 nm, while Dragon obtained larger particles, about 20 nm. We found that synthesis altered the chemical content of the extracts, particularly in lipid, protein, and tocopherol content, suggesting active involvement of Spirulina-derived biomolecules in nanoparticle formation. Antimicrobial assays showed slightly higher activity for Dragon AgNPs against *P. aeruginosa* (21 mm) and *S. enteritidis* (23 mm), with similar effects against *L. monocytogenes* and *S. aureus*. At 2.5 mg/mL, both samples protected human albumin from thermal denaturation more effectively (23.36% and 20.07%) than prednisolone (16.99%). Based on the obtained results, AgNPs from *Spirulina platensis* can be attributed as multifunctional agents with anti-inflammatory and antimicrobial activity.

## 1. Introduction

**Figure 1:**
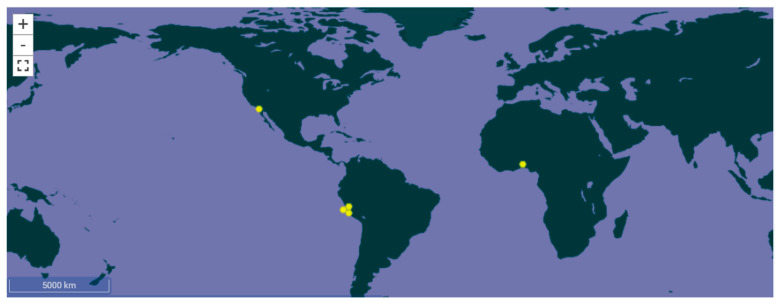
Global distribution map of *Arthrospira platensis* Gomont. Reproduced from GBIF.org Available online: https://www.gbif.org/species/3218108 (accessed on 7 September 2025), under the terms of the Creative Commons Attribution 4.0 License [5].

*Arthrospira platensis*, also known as *Spirulina*, is a common cyanobacteria that grows in both fresh- and saltwater environments of various kinds [1]. *Spirulina* spp. is classified in the kingdom *Bacillati*, phylum *Cyanobateriota* (blue-green algae), class *Cyanophyceae*, order *Spirulinaces*, and family *Spirulinaceae* [2]. With a 3.5-billion-year lifespan, *Spirulina* is also among the oldest living things on Earth [3]. *Spirulina*, a filamentous alga, consists of one or more cells and measures 200–500 μm in length and 5–10 μm in width. It can be found in a variety of habitats, but primarily grows in tropical and subtropical regions, alkaline lakes, and extreme environments like Antarctic ice lakes [4] (Figure 1).

The first factory for *Spirulina* processing was built in France in 1969. While over 50 species of *Spirulina* have been identified, only two—*Spirulina maxima* and *Spirulina platensis*—are cultivated extensively worldwide [6]. Microalgae must be grown in controlled industrial settings inland because of their small size, which prevents them from being gathered directly from their natural habitat [7]. For instance, even at 1% concentration, *Spirulina maxima* can be used as a biofertilizer to improve all aspects of plant growth and its chemical composition (carbohydrates, proteins, lipids, vitamins, and growth phytohormones), including antioxidant activity [8]. *Spirulina platensis* is widely acknowledged as safe for ingestion in a number of countries, including China, India, Canada, the EU, and Japan [9]. *Spirulina* is considered safe by the US Food and Drug Administration (FDA) and is the most widely consumed microalga in the EU and the US [10].

*Spirulina* contains a variety of amino acids and full protein, which makes up 60–70% of its dry weight. The amino acids alanine, glycine, glutamic acid, serine, aspartic acid, lysine, leucine, arginine, proline, threonine, valine, phenylalanine, isoleucine, and tyrosine are all present in considerable concentrations in the microalgae *Spirulina* [11]. The amount of amino acids, especially the amounts of lysine, histidine, and aspartic acid, may increase during fermentation [12]. Naturally dried Cuban *Spirulina* contains higher quantities of valine, leucine, and glutamic acid [13].

**Figure 2:**
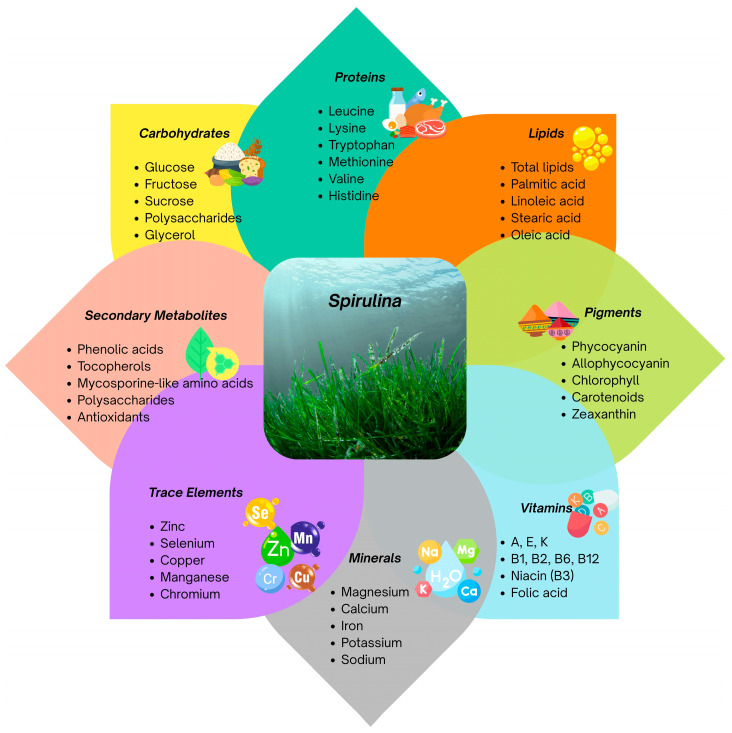
Main bioactive components of *Spirulina platensis* and their functional classification. Illustration created with Canva (www.canva.com).

*Spirulina* spp. contains a notable amount of polyunsaturated fatty acids (PUFAs), which constitute 1.5–2.0% of its total lipid content (Figure 2). *Spirulina* contains several predominant fatty acids, including 9,12,15-octadecatrienoic acid, hexadecanoic acid, 9,12-octadecadienoic acid, 4,7,10,13-hexadecatetranoic acid, 6,9,12-octadecatrienoic acid, 7,10,13-hexadecatrienoic acid, 9-hexadecenoic acid, 7,10-hexadecadienoic acid, 5,8,11,14,17-eicosapentaenoic acid, 6,9,12,15-octadecatetraenoic acid, and octadecanoic acid [13]. *Spirulina*’s list of fatty acids includes α-linolenic, palmitic, linoleic, γ-linolenic, omega-9, polyunsaturated, omega-3, and omega-6 fatty acids, as well as stearidonic acid, eicosapentaenoic acid, docosahexaenoic acid, and arachidonic acid [14,15].

*Spirulina* spp. has numerous applications in a range of medical conditions, such as immune system modulation, antioxidant properties, diabetes management, cardiovascular health, anticancer, prebiotic and probiotic properties, eye conditions, anti-anemic, neuro-protective, etc. [16]. *Arthrospira platensis* has been shown to reduce blood sugar [17] and LDL cholesterol [18].

Noble metal nanoparticles (NPs) have drawn a lot of attention in recent decades because of their growing uses in biological and pharmacological domains, such as drug delivery, photothermal therapy, radiotherapy, and imaging [19]. Because of their exceptional biological activity and distinct physicochemical properties, gold and silver nanoparticles (AgNPs) have been produced in the greatest quantities among them [20,21,22].

Green synthesis is the use of biological processes, including plants, algae, fungi, and bacteria, to produce materials, especially nanoparticles like AgNPs, in an environmentally benign manner [23]. This approach differs from conventional synthesis, which frequently uses hazardous chemicals and requires much energy. Green synthesis relies on sustainable, renewable materials while reducing waste and its impact on the environment. Utilizing renewable resources such as microorganisms and plant extracts, reducing waste production, and employing non-toxic solvents like ethanol or water are all cornerstones of green synthesis [24].

Furthermore, in contrast to the high-energy processes of conventional approaches, green synthesis is usually energy-efficient and frequently conducted in mild circumstances, such as room temperature or low heating. Additionally, biological molecules act as reducing and stabilizing agents during green synthesis, enhancing the final nanoparticles’ biological activity and biocompatibility [25]. On the other hand, substances like sodium borohydride or hydrazine, which are harmful to both human health and the environment, are commonly used in conventional synthesis techniques [26,27]. These techniques also frequently use much energy and produce much waste and hazardous byproducts, which go against the ideals of green chemistry. Furthermore, the more straightforward and economical one-step green synthesis is, the more accessible and sustainable than traditional procedures, which frequently require expensive and complicated equipment.

Phytochemicals frequently have a range of biological characteristics, such as antibacterial, anti-inflammatory, and antioxidant effects. These properties allow plant extracts to play catalytic and stabilizing roles in the creation of nanoparticles [28]. For example, plant extracts high in polyphenols have the ability to modulate the rate of reaction during the synthesis of AgNPs and are crucial in determining the size of the final nanoparticles. Additionally, flavonoids have the ability to adsorb onto nanoparticle surfaces, changing their characteristics for a range of uses [29].

Recent advances in green nanotechnology have enabled the synthesis of AgNPs using *Spirulina* as a reducing and stabilizing agent [30,31]. These biosynthesized AgNPs display potent antimicrobial activity, often superior to that of conventional antibiotics, due to their ability to disrupt microbial membranes and generate reactive oxygen species [32,33]. Moreover, the eco-friendly synthesis approach reduces cytotoxicity and environmental impact compared to chemical methods [34,35,36]. Recent advances in green nanotechnology have enabled the synthesis of AgNPs using *Spirulina* as a reducing and stabilizing agent [30,31]. These biosynthesized AgNPs display potent antimicrobial activity, often superior to that of conventional antibiotics, due to their ability to disrupt microbial membranes and generate reactive oxygen species [32,33]. Moreover, the eco-friendly synthesis approach reduces cytotoxicity and environmental impact compared to chemical methods [34,35,36].

AgNPs obtained using phycocyanin obtained from *Spirulina platensis* have been recently reported [37]. Gul et al. also synthesized *Spirulina*-mediated AgNPs [38] in order to investigate their anticoagulant and thrombolytic potential, as well as their biocompatibility and their potential in the degradation of toxic industrial dyes.

Harutyunyan et al. provided a comparative study of physicochemical properties and antibacterial potential of *Spirulina platensis* biomass and chemically synthesized AgNPs [39]. The authors concluded that *Spirulina*-derived AgNPs are synthesized as a low-cost, simple approach to producing stable AgNPs, compared to chemically obtained nanoparticles. Recently, Bej et al. reported biosynthesis and the antibacterial effect of AgNPs from aqueous extracts of *Spirulina* sp. and *Spirulina subsalsa* [40]. Rudi et al. also investigated the biocompatibility and physiological impacts of AgNPs, functionalized with *Spirulina* protein extract. The authors found that biofunctionalization of AgNPs modifies the behavior of nanoparticles, enhancing their biocompatibility while inducing minimal physiological stress [41].

To the best of our knowledge, there is no information available regarding a thorough analysis of the chemical composition of *Spirulina* extracts and how it changes following nanoparticle synthesis. Therefore, the present study aimed to use green methods for AgNP synthesis using two *Spirulina* (*Arthrospira platensis*) extracts and examine the variations in their chemical compositions. One of them, cultivated in a bioreactor in Bulgaria (near Varvara village), was studied after convective drying [42] and the other one was bought from the local market in Bulgaria (Dragon Superfoods).

## 2. Materials and Methods

### 2.1. Plant Material

Two samples of *Spirulina* were analyzed. The first one, cultivated and grown in a bioreactor in Bulgaria (near Varvara village), was studied after convective drying [42]. The conditions for growth and the habitat of *Arthrospira platensis* were described by G. Gentscheva et al. in the following steps: sowing, changing the habitat of the samples from the laboratory into the production conditions, and growing the samples into a large volume [43]. The laboratory bioreactor culture, harvested after 25 days of cultivation, was collected as fresh wet biomass and subsequently dried in a thin layer with transversely oriented airflow towards the product layer at 45 ± 2 °C and a relative humidity of the circulating air, on average, 10%. Reaching the sample’s constant mass indicates the end of the drying process. The second one was bought from the local market in Bulgaria (Dragon Superfoods). The biomass was provided as commercially packaged dried powder; according to the manufacturer’s specification, the product was obtained from algae harvested at the exponential growth phase.

The objective of this study is to analyze the NPs obtained from plant extracts from the two samples and to find any potential parallels or discrepancies in the samples’ chemical composition and their antimicrobial and anti-inflammatory potential.

### 2.2. Extract Preparation

Subsequently, 1 g of powdered Spirulina (*Arthrospira platensis*) was submerged in 10 mL of 80% ethanol and agitated at 40 °C for 40 min. The resultant leaf infusion was filtered using Whatman paper. Then, 1 mL of extract was mixed with 9 mL of a 10 mM AgNO_3_ solution. The mixture underwent stirring for 4 min at ambient temperature, resulting in a distinct color transition from pale yellow to dark brown.

### 2.3. Analytical Techniques for Characterization of the AgNPs

After NPs preparation, the solution was used for ATR, transmission electron microscopy (TEM), dynamic light scattering (DLS), and zeta potential.

#### 2.3.1. FTIR Spectra

ATR spectra were determined on a VERTEX 70 FT-IR spectrometer (Bruker Optics, Ettlingen, Germany). The spectra were collected from 600 cm^−1^ to 4000 cm^−1^ with a resolution of 4 nm and 32 scans. The instrument was equipped with a diamond attenuated total reflection (ATR) accessory (PIKE MIRacle™ Single Reflection ATR device, ZnSe crystal, Madison, WI, USA). The spectra were analyzed with the OPUS-Spectroscopy Software, Bruker (Version 7.0, Bruker, Ettlingen, Germany).

#### 2.3.2. TEM (Transmission Electron Microscopy)

The TEM images were taken on JEOL JEM 2100 HRTEM (200 kV, Tokyo, Japan). The nanoparticle suspension was deposited onto a copper grid covered by a carbon supporting film and dried in ambient atmosphere.

#### 2.3.3. Zeta Potential

A Brookhaven BI-200 goniometer with vertically polarized incident light at a wavelength l = 632.8 nm supplied by a He–Ne laser operating at 35 mW and equipped with a Brookhaven BI-9000 AT digital autocorrelator was utilized. The scattered light was measured for dilute aqueous dispersions in the concentration range 0.056–0.963 mg mL^−^^1^ at 25, 37, and 65 °C. Measurements were made at θ angles in the range of 50–130°. The system allows measurements of ζ-potential in the range from −200 mV to +200 mV. All analyses were performed in triplicate at 25 °C.

### 2.4. Chemical Composition

The oil was isolated from the seeds in a Soxhlet extractor using *n*-hexane [44]. Total protein, moisture, and ash content were determined according to AOAC (2016) [45]. Total carbohydrates were calculated as follows: 100—(% protein + % lipids + % water + % ash) (FAO, 2003) [46].

#### 2.4.1. Fatty Acid Composition

The fatty acid profile was determined using gas chromatography (GC) following transesterification of the obtained glyceride oils (ISO 12966-1:2014; ISO 12966-2:2017) [47,48]. The analysis utilized an Agilent 8860 system (Santa Clara, CA, USA) equipped with a flame ionization detector (FID) and a DB-Fast FAME capillary column (Agilent, USA) (30 m × 0.25 mm × 0.25 μm). The temperature program began at 70 °C (held for 1 min), then increased at 5 °C per minute until reaching 250 °C with a 3-min hold; the injector was maintained at 270 °C, and the detector at 300 °C. For compound identification, a standard 37-component FAME mixture (Supelco, Bellefonte, PA, USA) was analyzed under the same GC conditions.

#### 2.4.2. Tocopherol Composition

Tocopherols were determined by a high-performance liquid chromatograph Merck–Hitachi system (Burladingen, Germany) with Nucleosil Si 50-5 column (250 × 4 mm, particle size: 5 μm), and fluorescent detection (295 nm excitation and 330 nm emission). The operating conditions were as follows: the mobile phase was hexane: dioxane, 96:4 (*v*/*v*), and the flow rate was 1 mL/min (ISO 9936, 2016) [49].

### 2.5. Microbiological Tests

#### Test-Microorganisms

For the determination of antimicrobial activity of water and methanol extracts as test—microorganisms were used: nine pathogenic microorganisms (*Staphylococcus aureus* ATCC 25923, *Listeria monocytogenes* NBIMCC 8632, *Klebsiella* sp. (clinical isolate), *Enterococcus faecalis* ATCC 29212, *Escherichia coli* ATCC 8739, *Salmonella enteritidis* ATCC 13076, *Proteus vulgaris* ATCC 6380, *Pseudomonas aeruginosa* ATCC 9027, and *Candida albicans* NBIMCC 74), two spore-forming microorganisms (*Bacillus cereus* ATCC 14579, *Bacillus subtilis* ATCC 6633), five fungi (*Aspergillus niger* ATCC 1015, *Aspergillus flavus*, *Penicillium chrysogenum*, *Fusarium moniliforme* ATCC 38932, and *Mucor* sp.) and yeast (*Saccharomyces cerevisiae* ATCC 9763) from the collection of the Department of Microbiology at the University of Food Technologies, Plovdiv, Bulgaria, were selected for the antimicrobial activity test. Strains develop as follows: the yeast *S. cerevisiae* was cultured on MEA at 30 °C for 24 h. The fungi *A. niger*, *A. flavus*, *P. Chrysogenum*, *Mucor* sp., and *F. moniliforme* were grown on MEA at 30 °C for 7 days or until sporulation. *B. subtilis* and *B. cereus* were cultured on Luria–Bertani agar with glucose (LBG agar) at 30 °C for 24 h. *S. aureus*, *L. monocytogenes*, *K. pneumoniae*, *E. faecalis*, *E. coli*, *S. enteritidis*, *P. Vulgaris*, *P. aeruginosa*, and *C. albicans* were cultured on LBG agar at 37 °C for 24 h [50,51].

The agar-diffusion well method is used to determine the antimicrobial activity of the aqueous and methanolic extracts [52,53]. First, 18 mL is pre-melted, cooled to 40–45 °C, and infected with the specified test microorganism (1.0 × 10^6^ cfu/mL for spores of mold fungi and 1.0 × 10^8^ cfu/mL for viable cells of bacteria and yeast). Then, the LBG-agar medium is poured into Petri dishes (*d* = 9 cm), placed on a level surface. After spilling the infected culture medium, the Petri dishes were left for 1 h to solidify the agar. Using a cylindrical well puncher, 6 wells (*d* = 6 mm) were cut in the agar, and 60 μL each of the aqueous and methanolic extracts of each flour sample obtained from FF and SF were instilled in triplicate. The Petri dishes are thermostated at different temperature conditions (depending on the type of test microorganism) for 24/48 h. The presence and degree of antimicrobial activity were determined by measuring the diameter of the inhibition zones around the agar wells. High antimicrobial activity is reported for inhibition zones 18 mm or more; moderate inhibition zones are between 12 and 18 mm; low inhibition zones are up to 12 mm, or completely absent inhibition zones.

### 2.6. Albumin DenaturationInhibition Method

*Spirulina* extracts were prepared in 80% methanol; the hydromodulus was 1:10. A single extraction was performed in an ultrasonic bath at 40 °C for 40 min. The extract was filtered through filter paper, and the solvent was evaporated on a rotary vacuum evaporator at reduced pressure. A final concentration of 2.5 mg/mL was achieved by dissolving the dried *Spirulina* extracts in DMSO. The algae extracts were subjected to an in vitro albumin denaturation inhibition assay.

The anti-denaturation assay was performed according to Milusheva et al. [54]. The reaction mixture was prepared with 0.5 mL of 5% aqueous solution of human albumin (Albunorm20, Octapharma AG, Brussels, Belgium) and 0.2 mL of the solution of the extracts of chlorella, dissolved in DMSO with a concentration of 2.5 mg/mL and 2.5 mL of phosphate-buffered saline—PBS (pH 6.3). For the blank, a mixture of 2.5 mL buffer and 0.2 mL DMSO was used instead of the algae extracts, and the contents of the control test included 0.5 mL serum albumin and 2.5 mL buffer. The samples were incubated at 37 °C for 15 min, followed by heating at 80 °C for 30 min, then cooled for 5 min. The absorbance of the samples was measured spectrophotometrically at 660 nm (Cary 60 UV-Vis, Agilent Technologies, Santa Clara, CA, USA). The percentage of inhibition of protein denaturation (% IPD) was calculated according to the following Equation (1):

% *inhibition denaturation* = (Absorbance *control* − Absorbance *sample*)/(Absorbance *control*) × 100 (1)

The control represents 100% protein denaturation. A commercially available anti-inflammatory drug is used for comparison. Its anti-inflammatory effect is determined as the percentage of inhibition of albumin denaturation, following the same method as for the *Spirulina* extracts.

### 2.7. Statistical Analysis

The statistical analysis was performed using SPSS 23.0 software (SPSS Inc., Chicago, IL, USA). All experimental data were presented as mean ± SD (standard deviation). Statistically significant differences for multiple comparisons were performed using Duncan’s test (chemical content).

Statistical significance between two independent groups (antimicrobial) was analyzed by an independent sample Student’s *t*-test, and differences were considered significant at *p* < 0.05.

## 3. Results and Discussion

### 3.1. Characterization

**Figure 3:**
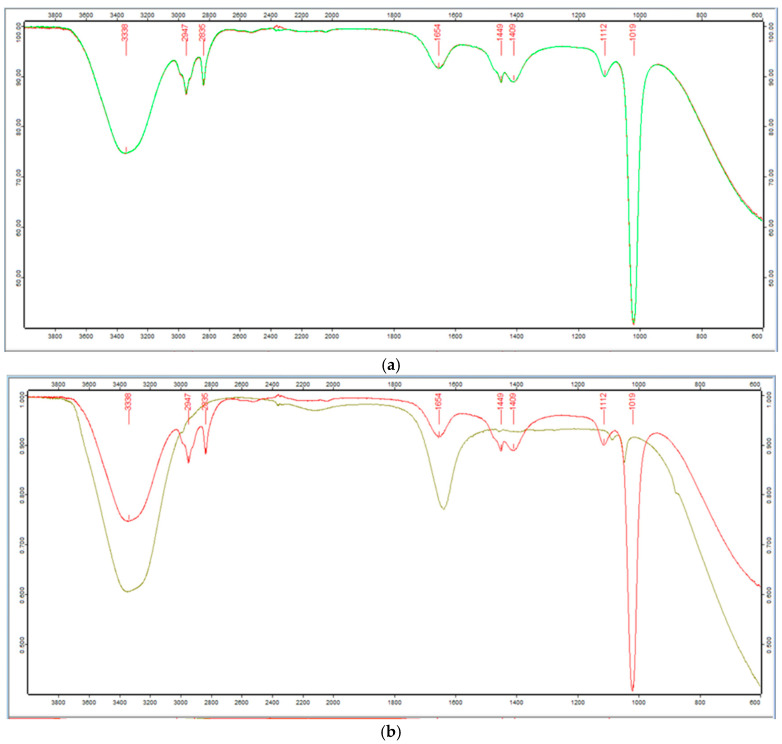
(**a**) ATR-spectra of *Spirulina* (Bioreactor) extract, (**b**) ATR spectra of *Spirulina* (Bioreactor) extract with ATR spectra of AgNPs obtained from the extract.

ATR analyses were conducted to discern potential biomolecules responsible for coating and stabilizing *Spirulina* leaf extracts, as well as the AgNPs synthesized from these extracts. In the ATR spectra of both *Spirulina* extracts can be observed characteristic peaks corresponding to different biomolecules, such as lipids (3020–2800 cm^−1^), proteins (1600–1500 cm^−1^), and polysaccharides (1300–1000 cm^−1^) [55] (Figure 3a).

The broad band around 3338 cm^−1^ at the wavenumber range 3680–3030 cm^−1^ could be attributed to -O-H vibrational stretching, but the shoulder at 3258 cm^−1^ corresponds to –NH group. The C-H vibrations of the alkyl groups are located in the ranges 2991–2927 cm^−1^ and around 2835 cm^−1^. These signals could be assigned to lipid and protein methylene vibrations [56]. The adsorption peaks in the regions around 1654 cm^−1^ could be assigned to –C=O stretches of aldehydes, ketones, Amide I of proteins, and carboxylate groups. These vibrations can be attributed to functional groups present in the proteins in both *Spirulina* samples. The band around 1449 cm^−1^ CH_3_ corresponds to out of plane lipids and protein vibrations. The functional groups in the samples’ carbohydrate components are responsible for the signals in the 1110–1000 cm^−1^ range [55]. The C-O, C-C, C-O-C, and C-O stretching of glycogen are represented by these bands [56].

In Figure 3, ATR spectra of *Spirulina* (Bioreactor) and AgNPs are compared. Some differences are observed in terms of the intensity of the bands and their changes in wavenumber.

A shift of the bands around 3341 cm^−1^ and 3274 cm^−1^ to 3353 cm^−1^ and 3258 cm^−1^, respectively, results from comparing the spectra of *Spirulina* (Bioreactor) extract with those of AgNPs, is observed (Figure 3b). In particular, the existence of functional hydroxyl groups (-OH, -COOH) in phenolic compounds, which are in charge of reducing Ag^+^ to Ag^0^, is confirmed by the broad –OH stretching band, which reaches its maximum at about 3350 cm^−1^. Additionally, this band’s intensity in the AgNPs’ spectra increased noticeably.

It appears that the pure sample extract’s spectrum only shows the bands around 2974 and 2835 cm^−1^ as shoulders around 2987, 2922, and 2852 cm^−1^. The shift to a lower wavenumber (1636 cm^−1^) and the decrease in the relative intensity of the peak at 1654 cm^−1^ indicated that amide and carboxyl groups were involved in the adsorption of NPs. This could be because of the interaction between the cation and the amide group, which is characterized by electron lone pairs over oxygen and nitrogen atoms.

The adsorption of NPs did not appear to have a substantial impact on this spectral area, which corresponds to the carbohydrate functional groups. The C–O, C–C, and C–OH stretching peaks in the AgNPs’ treated sample spectrum are shifted from 1113 cm^−1^ and 1018 cm^−1^, which indicate the CO stretching of the alcoholic group, to approximately 1085 cm^−1^ and 1045 cm^−1^ [57].

To establish the size and shape of the AgNPs obtained, BFTEM and Zeta potential were used.

### 3.2. TEM Micrographs

**Figure 4:**
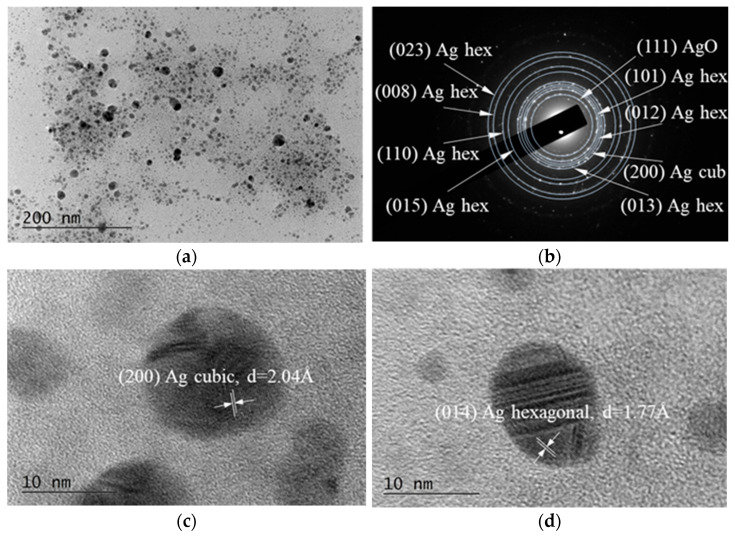
TEM images of the AgNPs obtained from the *Spirulina* (Bioreactor): (**a**) HRTEM of AgNPs from selected area; (**b**) SAED image of randomly selected Ag nanoparticle; (**c**) HRTEM of a cubic Ag; (**d**) HRTEM of a hexagonal Ag.

Bright Field TEM (BFTEM) micrographs, the corresponding Selected Area Electron Diffraction (SAED) pattern, and the High-Resolution TEM (HRTEM) image are presented in Figure 4.

It is established that truncated decahedral structures are often observed in Au and Ag nanoparticles produced by colloidal methods. It is worth noting that in the quantum size regime, truncation is the most common mechanism that nature chooses to reduce the total energy of the particles [58]. The emergence of elongated nanoparticles, multiple domain clusters, and irregular structures could be attributed to the preferential growth of decahedral nanoparticles or the coalescence of smaller nanoparticles.

The form and size of the silver accumulation are influenced by the reaction conditions, such as temperature, pH, extract volume, reactant concentration, and duration. These factors also control the size and gradation of the developing particles [59]. Nuclear openings may allow AgNPs smaller than 10 nm to pass through and engage with genetic material. These crystals are suitable for diagnostics and gene therapy, but they have genotoxic effects. The nanoparticle shape has been demonstrated to influence cytotoxicity; for instance, plate-shaped AgNPs exhibit greater toxicity than wire or spherical variants [60,61,62,63].

In our results, the individual NPs obtained from *Spirulina* (Bioreactor) have predominantly spherical-like form, apparently well faceted with a diameter of 5–50 nm (Figure 4). Some of the observed particles are slightly elliptical or irregular due to partial agglomeration or growth kinetics during the synthesis. A few larger particles or agglomerates are present, approaching 80–100 nm. Smaller, more uniformly dispersed nanoparticles (<10 nm) are visible in the upper portion of the image. There is moderate aggregation in some areas, with nanoparticles clustering together to form electronically denser regions.

The particles showed strong contrast against the background, indicating high electron density typical of metallic silver.

In order to determine the phase composition of the sample, SAED analysis was performed. The results reveal that the sample consists of three different phases, namely: hexagonal Ag, with lattice parameters a = 2.8862 Å, c = 10.0 Å [COD Entry #96-150-9195]; cubic Ag, with a = 4.071 Å, [COD Entry #96-150-9147]; and cubic AgO, a = 4.816 Å, [COD Entry #96-710-9247].

**Figure 5:**
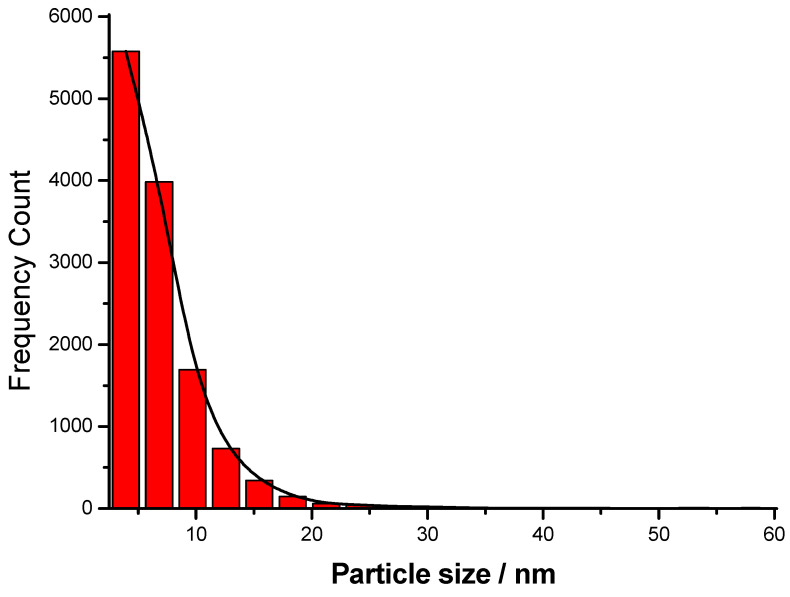
Size-distribution histogram of AgNPs from *Spirulina* (Bioreactor).

The histogram depicted in Figure 5 shows that the size distribution is right-skewed, with the majority of nanoparticles’ diameters below 10 nm. The dominance of small AgNPs (<10 nm) is typical for many chemical or green synthesis methods. The narrow size distribution and low polydispersity suggest controlled nucleation and growth conditions during synthesis. The presence of larger particles (outliers) reveals possible secondary nucleation or slight agglomeration. Such distributions are typical for the wet-chemical reduction methods of preparation, where growth kinetics lead to a primary population with a few larger crystals forming over time and reaching up to 55 nm in this case. The peak population lies between 4–8 nm, indicating that this is the most common particle size range. The calculated mean diameter of the AgNPs prepared in this study is about 6.8 nm.

**Figure 6:**
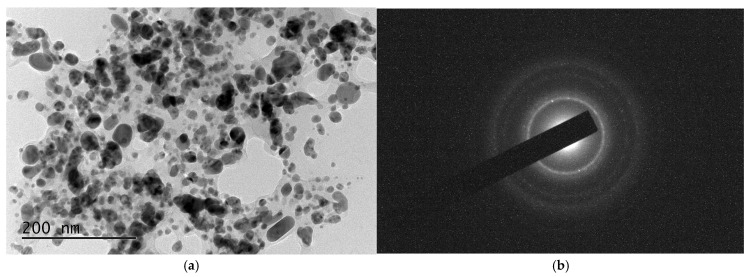
(**a**) HRTEM image of Ag nanoparticles synthesized from *Spirulina* (Dragon); (**b**) SAED image of randomly selected Ag nanoparticles.

The HRTEM image with 40 k resolution of AgNPs obtained from *Spirulina* (Dragon) is presented in Figure 6. As can be seen, the distribution appears polydisperse with a wide variety of particle sizes rather than a narrow distribution. Most particles are spherical or quasi-spherical, with a few showing elongated or elliptical morphologies. This suggests a mixture of growth kinetics during synthesis, possibly due to *Spirulina* extract components. A significant agglomeration is also evident, which is typical for metallic nanoparticles, and it can be explained by the fact that no strong capping or stabilizing agents were used during the synthesis, and by the large specific surface area.

**Figure 7:**
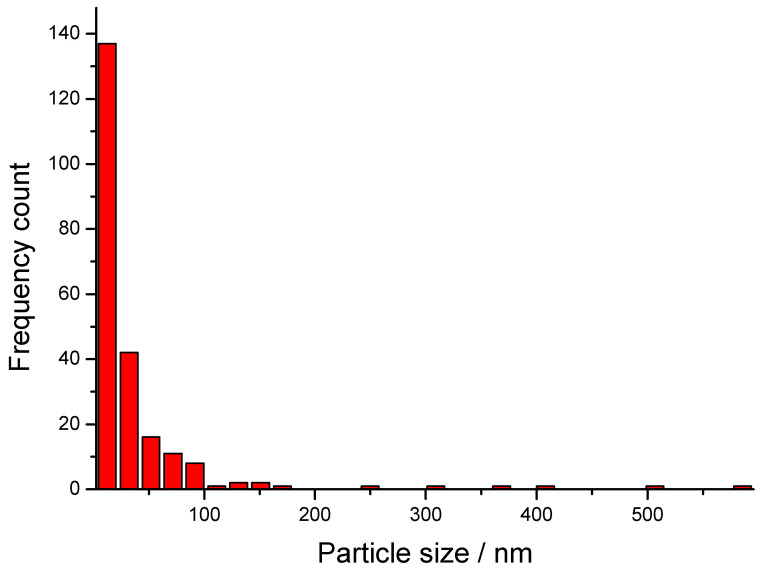
Size distribution of the AgNPs obtained from *Spirulina* (Dragon).

The particles range approximately from 10 nm to 60 nm in diameter (estimated using a 200 nm scale bar), which is in good agreement with the histogram presented in Figure 7. Most of the NPs fall in the 10–50 nm range, where the most dominant group of particles is with a size of 20 nm. A few bigger particles, up to 600 nm in size, are also present but comparatively uncommon due to the distribution’s right skew. This is typical in the green synthesis methods, where nucleation dominates early and the crystal growth is more variable.

SAED image presented in Figure 6b confirms the presence of ring patterns of the single face-centered cubic (fcc) crystalline spherical particles, with a preferential growth direction along the Ag (110), (200), (220), and (311) planes.

### 3.3. Zeta-Potential

**Figure 8:**
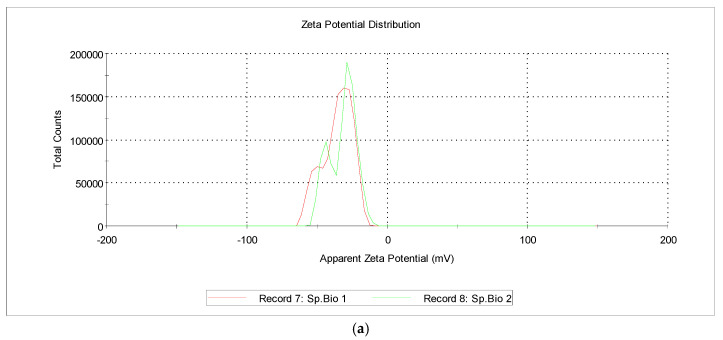

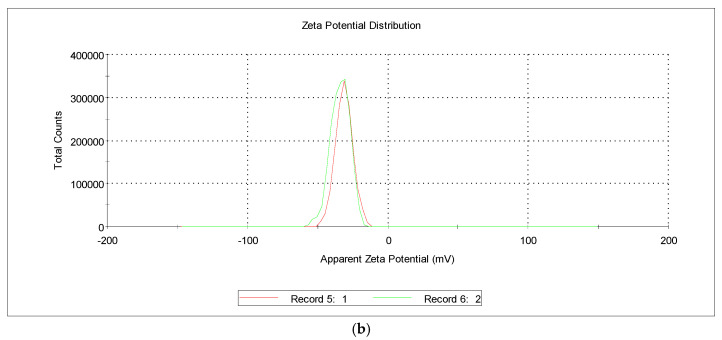
Zeta potential of AgNPs from *Spirulina* extracts (**a**) Bioreactor, (**b**) Dragon.

Flavonoids, tannins, saponins, phenolic acids, and other biomolecules on the surface of AgNPs serve as capping agents due to their negative electric charge. They typically possess a negative charge and generate repulsive forces that inhibit aggregation and maintain the stability of AgNPs in solution. Furthermore, the distribution of particle sizes has been influenced by AgNPs’ zeta potential. As depicted in Figure 8, AgNPs from *Spirulina* (Bioreactor) exhibited a zeta potential of −32.5 mV, while those from *Spirulina* (Dragon) exhibited −33.7 mV.

Khoshnamvand et al. previously reported that the zeta potential of AgNPs synthesized using *Allium ampeloprasum* L. leaf extract was −15.1 ± 5.89 mV [64]. Zeta potential of flaxseed extract was measured as −44.5 mV [65], −26 mV when synthesized from *E. camaldulensis* and −20 mV from *T. arjuna* [66].

Our results are fully consistent with the data reported by Abel-Fattah Salah Soror et al., who used phycocyanin from *Spirulina platensis* to synthesize AgNPs and measured a net negative charge of −26.32 mV for the zeta potential [37].

### 3.4. Chemical Composition Changes

The chemical composition of both *Spirulina* types (Dragon and Bioreactor) before and after the synthesis of AgNPs is given in Table 1.

**Figure 9:**
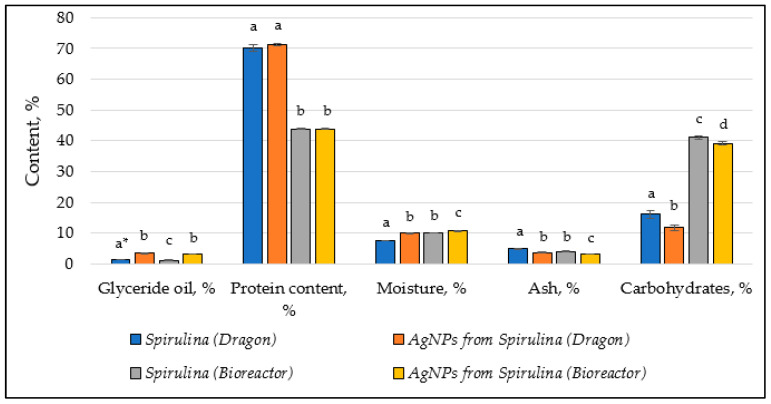
Changes in chemical composition of extracts and AgNPs obtained, *—Different letters in the components mean significant differences between the results (Duncan test, *n* = 3).

The data is schematically presented in the following graph (Figure 9). 

The synthesis of AgNPs significantly increased the glyceride oil content in *Spirulina* (Dragon) (from 1.37 to 3.37%) and *Spirulina* (Bioreactor) (from 1.12 to 3.10%). The increase in glyceride oil could suggest that the AgNPs’ synthesis process leads to enhanced lipid production. This might be due to the stress or biochemical changes induced by nanoparticle formation, which can affect metabolic pathways involved in lipid synthesis. The higher glyceride oil content might also reflect an alteration in the lipid metabolism of *Spirulina*, potentially influencing its nutritional value or functional properties. The increase in the lipid content could also be due to the interaction of lipids with silver ions during the synthesis process, as lipids may act as stabilizing agents for nanoparticles. Previous studies on the biogenic synthesis of AgNPs using *Spirulina maxima* suggest that biomolecules like lipids play a role in nanoparticle stabilization [67].

Both *Spirulina* extracts showed a small increase in protein content after the AgNPs’ formation: from 70.20 to 71.25% (*Spirulina* Dragon) and 43.80 to 43.90% (*Spirulina* Bioreactor), respectively. Its amount remains relatively stable, indicating that proteins are not heavily degraded during the process and may contribute to nanoparticle formation. Research on *Spirulina platensis* shows that proteins can act as reducing and capping agents in the green synthesis of nanoparticles [38].

There is an increase in moisture content in both *Spirulina* types after the synthesis of AgNPs. The rise in moisture could be linked to the interaction between *Spirulina* cells and the nanoparticles, possibly causing the cells to retain more water. This could be a result of the formation of a more hydrated extracellular matrix or changes in the structural integrity of the *Spirulina* cells due to nanoparticle interaction. While the moisture content of *Spirulina* (Bioreactor) is already higher than that of *Spirulina* (Dragon), AgNPs’ synthesis further exacerbates the moisture retention, which could impact the drying, storage, and processing characteristics of the *Spirulina* biomass. Similar observations have been noted in studies where *Spirulina* extracts were used for nanoparticle synthesis, leading to changes in their physical properties [30].

A noticeable decrease in the ash content in both extracts after the synthesis of AgNPs was also observed: from 4.84 to 3.69% (*Spirulina* Dragon) and from 3.97 to 3.19% (*Spirulina* Bioreactor), respectively. This reduction can be linked to the decrease in the amount of the total inorganic components, potentially due to their involvement in the synthesis process.

Both types of *Spirulina* show a decrease in carbohydrate content after AgNP treatment. In *Spirulina* (Dragon), the carbohydrate content decreases more significantly (from 16.05% to 11.69%), whereas in *Spirulina* (Bioreactor), the decrease is more moderate (from 41.11% to 39.09%). Carbohydrate levels drop significantly after AgNP synthesis, likely because carbohydrates are consumed as reducing agents in the process. Previous studies also confirm that polysaccharides in *Spirulina* are effective in reducing silver ions to form nanoparticles [30].

The fatty acid composition of the glyceride oils isolated from the two types of *Spirulina* (Dragon and Bioreactor) before and after the synthesis of AgNPs is presented in Table 2.

The fatty acid profiles of *Spirulina* (Dragon) and *Spirulina* (Bioreactor), before and after AgNP synthesis, reveal notable changes. Palmitic acid is the main fatty acid in all examined samples (30.8–63.8%), followed by oleic (5.0–23.8%), heptadecenoic (3.7–17.2%), heptadecanoic (2.7–12.6%), and stearic (3.7–13.9%) acids. A dramatic increase is observed in the content of palmitic acid in *Spirulina* (Dragon) after AgNP synthesis (from 30.8% to 63.8%), indicating a significant accumulation of this major saturated fatty acid (SFA). In *Spirulina* (Bioreactor), however, the content decreases slightly (from 42.5% to 37.0%). Oleic acid shows distinct changes in its content after the synthesis of AgNPs. It decreases slightly, from 5.7% to 5.0%, in *Spirulina* (Dragon), indicating a minor reduction during the synthesis process. On the other hand, in *Spirulina* (Bioreactor), the decrease is more pronounced, dropping from 23.8% to 12.5%. This suggests that oleic acid in *Spirulina* (Bioreactor) may play a more active role in the synthesis or stabilization of AgNPs. The content of heptadecenoic acid is significantly reduced in *Spirulina* (Dragon) after the synthesis of AgNPs (from 17.2% to 5.5%), whereas *Spirulina* (Bioreactor) showed an increase (from 3.7% to 12.1%). Regarding the stearic acid, it is observed that both variants of *Spirulina* showed a reduction in its content after synthesis, with a more pronounced decrease in *Spirulina* (Bioreactor) (from 13.9% to 5.6%). In *Spirulina* (Bioreactor), the content of caprylic acid increases after AgNP synthesis (from 0.9% to 1.6%), while it decreases slightly in *Spirulina* (Dragon) (from 0.6% to 0.4%). This suggests that the synthesis process may influence the shorter-chain fatty acids differently in the two variants. *Spirulina* (Bioreactor) showed a notable increase in linoleic acid (from 0.2% to 3.8%), while its amount in *Spirulina* (Dragon) remains relatively stable. On the other hand, *Spirulina* (Dragon) shows a slight increase in the content of α-linolenic acid (from 0.4% to 0.7%), while it is absent in *Spirulina* (Bioreactor).

The synthesis of AgNPs appeared to significantly alter the fatty acid composition of *Spirulina*, with notable differences between the two variants. These changes could be due to the interaction of fatty acids with silver ions during the synthesis process, as fatty acids may act as reducing or stabilizing agents. The increase in certain PUFAs, such as linoleic acid in *Spirulina* (Bioreactor), could enhance its nutritional and functional properties, while the accumulation of SFAs like palmitic acid in *Spirulina* (Dragon) may affect its stability and bioactivity.

Studies on the green synthesis of AgNPs using *Spirulina* extracts have shown that biomolecules, including fatty acids, play an important role in the reduction and stabilization of nanoparticles. Research on *Spirulina platensis* highlights the involvement of fatty acids in nanoparticle synthesis and their potential impact on the bioactivity of the final product [38,67].

**Figure 10:**
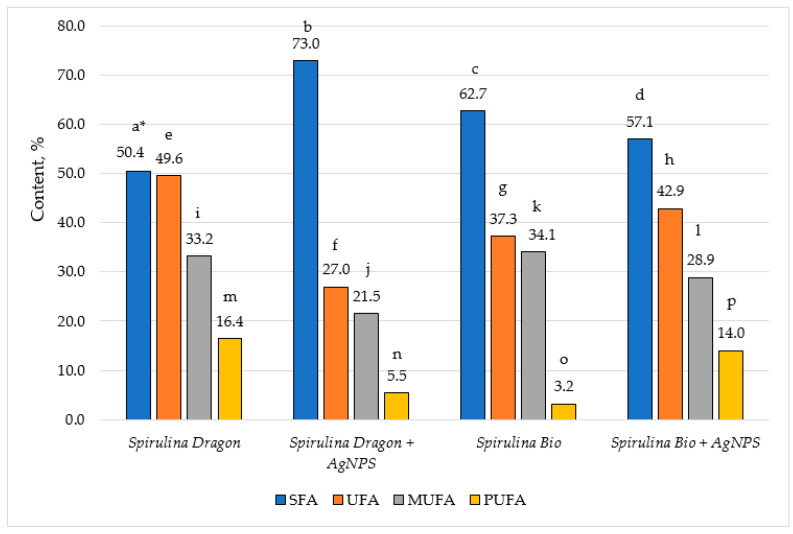
Content of saturated (SFA), unsaturated (UFA), mono- (MUFA), and polyunsaturated fatty acids (PUFA). *—Different letters in the fatty acid groups mean significant differences between the results (Duncan test, *n* = 3).

The content of the saturated (SFA), unsaturated (UFA), mono- (MUFA), and polyunsaturated fatty acids (PUFA), is depicted in Figure 10.

The data showed that the content of the SFA in all samples is over 50%: from 50.4% in *Spirulina* (Dragon) to 73.0% in the same species after the synthesis of AgNPs, while the amount of the UFA was lower: from 27.0% to 49.6%. The ratio of SFA to UFA in *Spirulina* (Dragon) is almost 1:1, while in *Spirulina* (Bioreactor) it is almost 2:1. The synthesis of AgNPs induces specific modifications in the fatty acid profile of the *Spirulina* samples. For *Spirulina* (Dragon), the SFA increased markedly from 50.4% to 73.0% following nanoparticle synthesis, while the UFA decreased considerably, from 49.6% to 27.0% overall, with both monounsaturated (MUFA: 33.2% to 21.5%) and polyunsaturated fractions (PUFA: 16.4% to 5.5%) being significantly reduced. This decrease suggests that reactive species generated during the AgNP formation preferentially oxidize unsaturated bonds, converting more labile fatty acids into saturated ones; a mechanism that is consistent with redox- and oxidation-related transformations observed during the biogenic synthesis of nanoparticles from *Spirulina maxima* [67]. In contrast, the *Spirulina* (Bioreactor) variant demonstrates a different pattern: its SFA content slightly decreases from 62.7% to 57.1% after AgNP synthesis, and its overall UFA content increases from 37.3% to 42.9%. Notably, this increase in UFA is attributable to a pronounced augmentation in the PUFA fraction (rising from 3.2% to 14.0%), even though MUFA decreases from 34.1% to 28.9%. Such contrasting behaviors suggest that the interaction between the silver ions and the bioactive compounds in *Spirulina* leads to differential oxidative or structural modifications in the lipid matrices of these variants. This indicates that the bioactive compounds in *Spirulina* (Bioreactor), containing differing protein and polysaccharide content, may protect the polyunsaturated fatty acids during metal ion reduction. What is more, the diverse responses between these *Spirulina* variants could be attributed to their biochemical differences that modulate the redox balance and influence nanoparticle formation, paralleling the bio-stimulant effects of algae polysaccharides reported for *Spirulina platensis* in NP synthesis processes [68].

Table 3 shows the results about the tocopherol composition of the two types of *Spirulina* (Dragon and Bioreactor) before and after the synthesis of AgNPs.

The data reveal significant changes in the tocopherol composition of *Spirulina* (Dragon) and *Spirulina* (Bioreactor) after their use in synthesizing AgNPs. The total tocopherol content declines substantially in both variants, dropping from 2432 mg/kg to 92.4 mg/kg in *Spirulina* (Dragon) and from 102 mg/kg to 42 mg/kg in *Spirulina* (Bioreactor), indicating a loss of antioxidant capacity. These changes suggest that tocopherols play an active role in NP synthesis, potentially acting as stabilizing or reducing agents, but their reduction impacts the overall antioxidant properties of *Spirulina*. This transformation highlights variant-specific differences and may have implications for the nutritional and functional properties of *Spirulina* after AgNP synthesis. The main tocopherol representative in *Spirulina* (Dragon) is α-tocopherol (being 84.1% of the total tocopherol content), followed by γ-tocopherol (7.4%). Other constituents detected in this sample are α-tocotrienol (3.8%), γ-tocotrienol (3.2%), and β-tocopherol (1.5%). After the synthesis of AgNPs, the only identified tocopherol in the sample is α-tocopherol, while the other isomers are completely degraded. On the other hand, *Spirulina* (Bioreactor) has a completely different tocopherol composition, with α-tocotrienol being the major one (66.1%). The other detected component is γ-tocotrienol with a content of 33.9%. After the synthesis of AgNPs is also observed a degradation of γ-tocotrienol, leaving α-tocotrienol the only present component in the fraction.

The observed changes in tocopherol composition during the synthesis of AgNPs using *Spirulina* can be attributed to the biochemical interactions between tocopherols and silver ions. Tocopherols, particularly α-tocopherol and α-tocotrienol, are known for their antioxidant properties, which enable them to act as reducing agents in nanoparticle synthesis. This process involves the donation of electrons by tocopherols to silver ions, facilitating their reduction to metallic silver and the formation of NPs [69]. The dominance of α-tocopherol in *Spirulina* (Dragon) and α-tocotrienol in *Spirulina* (Bioreactor) after synthesis suggests that these specific tocopherols play a pivotal role in stabilizing the nanoparticles. Their molecular structures make them effective capping agents, preventing aggregation and ensuring uniformity of the nanoparticles [69,70].

The drastic reduction in total tocopherol content in both *Spirulina* may suggest the extensive utilization of these compounds during synthesis. This decrease could impact the antioxidant capacity of *Spirulina*, as tocopherols are key contributors to its ability to neutralize free radicals.

### 3.5. Antimicrobial Activity

Antimicrobial activity was determined by measuring the diameter of the inhibition zones around the wells at 24 and 48 h of incubation (Table 4).

The two *Spirulina* extracts, as well as methanol used as a solvent for the samples, did not show any inhibitory effect against all pathogenic and saprophytic microorganisms tested.

**Figure 11:**
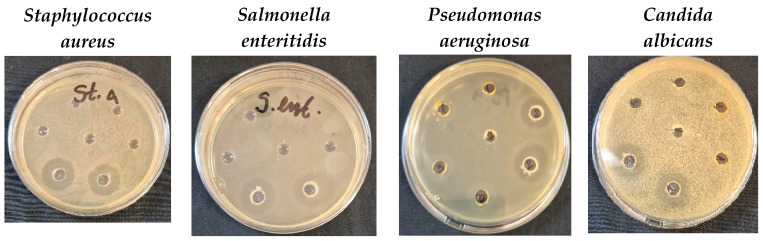
Selected petri dish photos of antimicrobial and antifungal activity assay of AgNPs from *Spirulina*.

The tested AgNPs from *Spirulina* showed low antimicrobial activity against *Klebsiella* sp., the yeast *S. cerevisiae*, and all fungi used in this study. No inhibitory effect was found against the fungi *Mucor* sp. On the other hand, the obtained results showed that Gram-positive bacteria, namely *S. aureus*, *L. monocytogenes*, as well as Gram-negative bacteria, namely *P. aeruginosa* and *S. enteritidis*, are more sensitive to AgNPs from both *Spirulina* samples, with an inhibition zone of more than 18 mm being established (Figure 11). Moderate antimicrobial activity of AgNPs was established against pathogenic *E. faecalis*, *E. coli*, *P. Vulgaris*, and spore-forming microorganisms *B. cereus* and *B. subtilis*. In the fungus *Candida albicans*, the antimicrobial effect is moderate, but in the zones of inhibition, there is a presence of single colonies.

Numerous complex factors, such as the zeta potential on the membrane, the lipophobicity of the cell membrane, the thickness of the membrane and its surrounding layers, and the chemical composition of the antimicrobial drug, greatly influence the permeability of the cell membranes of Gram-positive and Gram-negative bacterial strains, which accounts for this difference in activity [71].

Various studies have demonstrated that *Spirulina* extracts exhibit inhibitory effects against a range of bacterial and fungal pathogens. For example, methanolic and ethanolic extracts of *Spirulina* have shown activity against *Escherichia coli*, *Staphylococcus aureus*, and *Candida albicans* [72,73].

Previously obtained results confirmed our results. Soror et al. found no inhibition zones at low concentrations of AgNPs or the aqueous *Spirulina platensis* extract. However, at high concentrations, inhibition zones, especially AgNPs, were more potent for all tested microorganisms than their positive controls, with particular reference to *Staphylococcus aureus* [37].

Our results are consistent with those obtained by Harutyunyan et al., who found a very good antimicrobial activity of AgNPs from *Spirulina platensis* against Gram-positive (*Enterococcus hirae* and *Staphylococcus aureus*) and Gram-negative (*Pseudomonas aeruginosa* and *Salmonella typhimurium*) bacteria [39].

Overall, *Spirulina*-based antimicrobial agents, including extracts and AgNPs, offer a promising way for natural and sustainable antimicrobial therapies, especially in the face of rising antibiotic resistance.

### 3.6. Inhibition of Albumin Denaturation Results

Protein denaturation occurs in inflammatory conditions [35]. The inflammatory process can be acute and chronic. This process disrupts the biological functions of proteins and destroys their secondary and tertiary structures [36,74]. There are cases where the reduction of inflammatory activity is associated with the inhibition of protein denaturation [35]. The ability of a substance or therapy to reduce inflammation is associated with an anti-inflammatory effect. To control the inflammatory process, nonsteroidal and steroidal anti-inflammatory drugs are developed and used, but they also have concomitant side effects [75,76,77].

An alternative or concomitant therapy to conventional anti-inflammatory drugs could be various plant extracts based on the biologically active substances they contain and their potential therapeutic effect [78]. The inhibition of albumin denaturation assay has been used in a large number of studies conducted in recent years to examine the anti-inflammatory properties of extracts from different plant sections. The plant species studied are diverse and include *Ficus racemosa*, *Elaeocarpus tectorius* (Lour.) Poir, *Peltophorum pterocarpum*, *Mikania scandens* (L.), *Aidia genipiflora*, *Arthrophytum scoparium*, *Cajanus cajan* (Gungo), *Cinnamomum zeylanicum* (Cinnamon), *Cordia alba* (Duppy cherry), *Mangifera indica* (Julie mango), *Tecoma stans* (Jamaican lilac), etc. [79,80,81,82,83].

**Figure 12:**
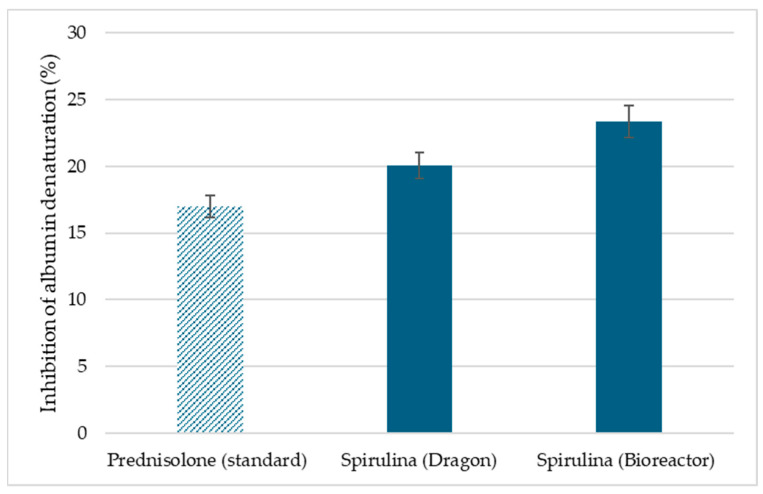
Inhibition of albumin denaturation by *Spirulina* extracts (Bioreactor and Dragon) compared with prednisolone. Results are expressed as mean ± SD (*n* = 3).

A rapid and inexpensive method was used to evaluate the anti-inflammatory activity of *Spirulina* (Bioreactor) and *Spirulina* (Dragon) extracts, and it was compared with an anti-inflammatory drug, prednisolone. The results are presented in Figure 12. At 2.5 mg/mL, *Spirulina* (Bioreactor) extract inhibited albumin denaturation by 23.36 ± 0.48%, *Spirulina* (Dragon) by 20.07 ± 0.57%, which were higher than the standard prednisolone (16.99 ± 0.48%).

The demonstrated in vitro anti-inflammatory properties of the algae extracts are most likely due to the substances contained in them that stabilize the protein molecule and contribute to the inhibition of thermal denaturation [36,74].

## 4. Conclusions

The present article demonstrates the synthesis of AgNPs from two *Spirulina platensis* extracts and variations in their chemical content after the synthesis of AgNPs. Our results indicate that AgNPs derived from the two extracts differ in morphology and zeta potential, as revealed by TEM and ATR-FTIR analyses. These variations were associated with differences in the chemical profiles of the extracts, particularly in their fatty acids, tocopherols, and protein content. The observed compositional alterations showed that *Spirulina platensis* is used as a reducing and stabilizing agent in the green synthesis of silver nanoparticles, but that it also has specific effects on the biochemical integrity of the particles. Based on the chemical composition of the extracts, results showed better antimicrobial and anti-inflammatory potential of the two types of NPs. AgNPs from the commercial *Spirulina* extract showed better activity against *P. aeruginosa* and *S. enteritidis*, and both types exhibited comparable effects against *L. monocytogenes* and *S. aureus*. Both AgNP samples demonstrated better anti-inflammatory activity in protection of protein denaturation than the commercial drug prednisolone. Overall, the results indicate the importance of algae origin in green nanoparticle synthesis, providing future directions for specific biomedical or nutraceutical applications.

## Figures and Tables

**Table 1:** Chemical composition of *Spirulina platensis’* extracts and AgNPs obtained.

Chemical Composition	*Spirulina* (Dragon)	AgNPs from *Spirulina* (Dragon)	*Spirulina* (Bioreactor)	AgNPs from *Spirulina* (Bioreactor)
Glyceride oil, %	1.37 ± 0.05 ^a^ *	3.37 ± 0.17 ^b^	1.12 ± 0.12 ^c^	3.10 ± 0.10 ^b^
Protein content, %	70.20 ± 1.00 ^a^	71.25 ± 0.50 ^a^	43.80 ± 0.20 ^b^	43.90 ± 0.10 ^b^
Moisture, %	7.54 ± 0.10 ^a^	9.95 ± 0.15 ^b^	10.00 ± 0.10 ^b^	10.72 ± 0.12 ^c^
Ash, %	4.84 ± 0.25 ^a^	3.69 ± 0.15 ^b^	3.97 ± 0.21 ^b^	3.19 ± 0.13 ^c^
Carbohydrates, %	16.05 ± 1.40 ^a^	11.69 ± 0.80 ^b^	41.11 ± 0.58 ^c^	39.09 ± 0.52 ^d^

*—Different letters in a row mean significant differences between the results (Duncan test, *n* = 3).

**Table 2:** Fatty acid composition of glyceride oil extracted from the analyzed samples.

Fatty Acids, %		*Spirulina* (Dragon)	AgNPs from *Spirulina* (Dragon)	*Spirulina* (Bioreactor)	AgNPs from *Spirulina* (Bioreactor)
C 8:0	caprylic	0.6 ± 0.1 ^a^ *	0.4 ± 0.1 ^b^	0.9 ± 0.2 ^c^	1.6 ± 0.3 ^d^
C 10:0	capric	-	-	0.2 ± 0.0 ^a^	0.5 ± 0.1 ^b^
C 12:0	lauric	-	-	0.3 ± 0.1 ^a^	1.0 ± 0.2 ^b^
C 14:0	myristic	1.3 ± 0.3 ^a^	0.9 ± 0.2 ^a^	1.4 ± 0.1 ^a^	1.5 ± 0.2 ^a^
C 14:1	myristoleic	3.6 ± 0.4 ^a^	5.3 ± 0.3 ^b^	2.7 ± 0.2 ^c^	1.1 ± 0.1 ^d^
C 15:0	pentadecanoic	-	-	0.2 ± 0.0 ^a^	0.6 ± 0.1 ^b^
C 15:1	pentadecenoic	1.9 ± 0.3 ^a^	0.2 ± 0.0 ^b^	0.9 ± 0.2 ^c^	1.5 ± 0.4 ^a^
C 16:0	palmitic	30.8 ± 0.5 ^a^	63.8 ± 0.5 ^b^	42.5 ± 0.4 ^c^	37.0 ± 0.3 ^d^
C 16:1	palmitoleic	4.8 ± 0.3 ^a^	5.5 ± 0.3 ^b^	2.3 ± 0.2 ^c^	1.7 ± 0.2 ^d^
C 16:2	7,10-hexadecadienoic	11.0 ± 0.4 ^a^	2.8 ± 0.2 ^b^	2.0 ± 0.1 ^c^	7.1 ± 0.2 ^d^
C 17:0	heptadecanoic	12.6 ± 0.5 ^a^	3.9 ± 0.3 ^b^	2.7 ± 0.2 ^c^	8.4 ± 0.4 ^d^
C 16:3	7,10,13- hexadecatrienoic	3.7 ± 0.2 ^a^	1.0 ± 0.1 ^b^	0.7 ± 0.1 ^c^	2.4 ± 0.2 ^d^
C 17:1	heptadecenoic	17.2 ± 0.5 ^a^	5.5 ± 0.3 ^b^	3.7 ± 0.2 ^c^	12.1 ± 0.3 ^d^
C 18:0	stearic	4.7 ± 0.3 ^a^	3.7 ± 0.2 ^b^	13.9 ± 0.4 ^c^	5.6 ± 0.2 ^d^
C 18:1	oleic	5.7 ± 0.3 ^a^	5.0 ± 0.4 ^a^	23.8 ± 0.5 ^c^	12.5 ± 0.3 ^d^
C 18:2	linoleic	0.7 ± 0.2 ^a^	0.6 ± 0.1 ^a^	0.2 ± 0.0 ^c^	3.8 ± 0.3 ^d^
C 18:3 n-3	α-linolenic	0.4 ± 0.1 ^a^	0.7 ± 0.2 ^a^	-	-
C 18:4	stearidonic	-	-	0.3 ± 0.1 ^a^	0.7 ± 0.2 ^b^
C 20:0	arachidic	0.2 ± 0.0 ^a^	0.2 ± 0.0 ^a^	0.3 ± 0.1 ^a^	-
C 20:1	eicosenoic	-	-	0.7 ± 0.2	-
C 20:4	arachidonic	0.6 ± 0.1 ^a^	0.4 ± 0.1 ^a^	-	-
C 22:0	behenic	0.2 ± 0.0 ^a^	0.1 ± 0.0 ^b^	0.1 ± 0.0 ^b^	0.7 ± 0.2 ^c^
C 24:0	lignoceric	-	-	0.2 ± 0.0 ^a^	0.2 ± 0.0 ^a^

*—Different letters in a row mean significant differences between the results (Duncan test, *n* = 3).

**Table 3:** Tocopherol content of glyceride oil extracted from the analyzed samples.

Tocopherols	*Spirulina* (Dragon)	AgNPs in *Spirulina* (Dragon)	*Spirulina* (Bioreactor)	AgNPs in *Spirulina* (Bioreactor)
α-tocopherol, %	84.1 ± 0.5 ^a^ *	100 ± 0.0 ^b^	-	-
α-tocotrienol, %	3.8 ± 0.2 ^a^	-	66.1 ± 0.4 ^b^	100 ± 0.0 ^c^
β-tocopherol, %	1.5 ± 0.1	-	-	-
γ-tocopherol, %	7.4 ± 0.4	-	-	-
γ-tocotrienol, %	3.2 ± 0.2 ^a^	-	33.9 ± 0.3 ^b^	-
Total tocopherol content, mg/kg	2432 ± 30 ^a^	92.4 ± 10 ^b^	102 ± 12 ^b^	42 ± 6 ^c^

*—Different letters in a row mean significant differences between the results (Duncan test, *n* = 3).

**Table 4:** Antimicrobial activity of AgNPs from *Spirulina* extracts.

Tested Microorganisms	Inhibition Zones, mm			
	AgNPs from *Spirulina* (Dragon)		AgNPs from *Spirulina* (Bioreactor)	
	24 h	48 h	24 h	48 h
*Staphylococcus aureus*	18 ± 0.0 ^a^	18 ± 0.0 ^a^	19 ± 1.0 ^a^	19 ± 1.0 ^a^
*Listeria monocytogenes*	20 ± 0.0 ^a^	20 ± 0.0 ^a^	20 ± 0.0 ^a^	20 ± 0.0 ^a^
*Klebsiella* sp.	8.5 ± 0.5 ^a^	8.5 ± 0.5 ^a^	10 ± 0.0 ^a^	10 ± 0.0 ^a^
*Enterococcus faecalis*	17 ± 0.0 ^a^	17.5 ± 0.5 ^a^	16 ± 0.0 ^a^	17 ± 0.0 ^b^
*Escherichia coli*	17 ± 0.0 ^a^	17 ± 0.0 ^a^	17 ± 1.0 ^a^	17.5 ± 0.5 ^b^
*Salmonella enteritidis*	23 ± 1.0 ^a^	23 ± 1.0 ^a^	21 ± 1.0 ^a^	21 ± 1.0 ^a^
*Proteus vulgaris*	17.5 ± 0.5 ^a^	17.5 ± 0.5 ^a^	15 ± 1.0 ^a^	17 ± 0.0 ^b^
*Pseudomonas aeruginosa*	19 ± 1.0 ^a^	23 ± 1.0 ^a^	17.5 ± 0.5 ^a^	19 ± 1.0 ^b^
*Candida albicans*	17.5 ± 0.5 ^a^	17.5 ± 0.5 ^a^	16.5 ± 0.5 ^a^	16.5 ± 0.5 ^a^
*Bacillus* *cereus*	14.5 ± 0.5 ^a^	14.5 ± 0.5 ^a^	15 ± 1.0 ^a^	15 ± 1.0 ^a^
*Bacillus* *subtilis*	12.5 ± 0.5 ^a^	12.5 ± 0.5 ^a^	12 ± 0.0 ^a^	12 ± 0.0 ^a^
*Saccharomyces cerevisiae*	9 ± 0.0 ^a^	9 ± 0.0 ^a^	8.5 ± 0.5 ^a^	8.5 ± 0.5 ^a^
*Aspergillus niger*	8 ± 0.0 ^a^	-	8 ± 0.0 ^a^	-
*Aspergillus flavus*	8 ± 0.0 ^a^	8 ± 0.0 ^a^	10 ± 0.0 ^a^	10 ± 0.0 ^a^
*Penicillium chrysogenum*	10 ± 0.0 ^a^	10 ± 0.0 ^a^	11 ± 0.0 ^a^	10.5 ± 0.5 ^a^
*Mucor* sp.	-	-	-	-
*Fusarium moniliforme*	8 ± 0.0 ^a^	-	8 ± 0.0 ^a^	-

Values are expressed as mean ± SD (*n* = 3). Letter a indicates no statistically significant difference (*p* > 0.05), while letter b indicates statistically significant differences between the two samples at the same incubation time (Student’s *t*-test, *p* < 0.05).

## Data Availability

The original contributions presented in this study are included in the article material. Further inquiries can be directed to the corresponding author(s).
